# Distributed spatial awareness for robot swarms

**DOI:** 10.1007/s10514-025-10228-1

**Published:** 2025-11-22

**Authors:** Simon Jones, Sabine Hauert

**Affiliations:** 1 Department of Engineering Maths, University of Bristol, Queens Road, Bristol, BS8 1QU UK; 2 Bristol Robotics Laboratory, T-Block, UWE, Bristol, BS16 1QY UK

**Keywords:** Swarm, Intralogistics, Shape formation, Gaussian belief propagation

## Abstract

Building a distributed spatial awareness within a swarm of locally sensing and communicating robots enables new swarm algorithms. We use local observations by robots of each other and Gaussian belief propagation message passing combined with continuous swarm movement to build a global and distributed swarm-centric frame of reference. With low bandwidth and computation requirements, this shared reference frame allows new swarm algorithms. We characterise the system in simulation and demonstrate two example algorithms, then demonstrate reliable performance on real robots with imperfect sensing.

## Introduction

Swarm robotics, inspired by swarms in nature, has the potential for resilient, robust, and redundant solutions to a wide range of problems such as mapping, logistics, search and rescue, disaster recovery, and environmental monitoring. Many relatively simple and cheap robots, each following simple rules, with local interactions between themselves and the environment are capable of producing a desired emergent swarm-level behaviour (Schranz et al., 2020; Jones et al., 2016; Crosscombe et al., 2017; Birattari et al., 2019; Tzoumas et al., 2023; Jones et al., 2019).

There are many areas of swarm algorithm design where access to global information would be useful, but unless that information is inferred or constructed through purely local interactions, it does not fit within the distributed swarm paradigm. One example is spatial awareness, by which we mean that an agent within the swarm is aware of its own location with respect to the swarm as a whole; the swarm shares a spatial reference frame. The availability of a completely distributed, completely local, low cost, shared reference frame would open up algorithmic approaches previously not possible. By using Gaussian belief propagation, we can construct this within a swarm of robots based only on local observation and messaging. Robots move around, constructing a distributed, size-limited factor graph of observations of other robots and odometry information. Message passing within and between neighbouring robots results in convergence on a shared frame of reference; each robot knows where it is in relation to it. To demonstrate the potential of this, we focus on two applications, shape formation and logistics, and follow up with application to a swarm of real robots.

Swarm shape formation is a proxy for a number of real-world problems such as search and rescue or emergency communication. Many problems rely on the swarm maintaining a coherent shape or coverage of particular areas. This has been tackled in swarm robotics in a number of ways which in their essence involve the construction of a frame of reference, or coordinate system. These systems often rely on robots transitioning to a static state to serve as anchors for further extensions to the shape and coordinate system, or unrealistic assumptions of position knowledge.

The use of swarms for intralogistics is an emerging area, where perhaps we can move beyond the lab into real-world applications. With our DOTS (Jones et al., 2022) robots we aim to demonstrate a simple but functional application. Specifically, we consider the potential for small scale out-of-the-box solutions for everyday environments; simply delimit an area of floor and add robots and small cargo carriers. Users would download an app to their phone and use this to call for a carrier to deposit an item. Via Bluetooth, any robot within range would respond and provide the carrier and take it and the item to be stored. To retrieve the item, the user would use the app again, robots would talk with their neighbours until a robot with recent knowledge of the item heard, which would pick up the carrier and take it to the user. Even simple random walk algorithms are capable of effective retrieval in logistics applications.

We describe the implementation of a system, which we call Distributed Spatial Awareness (DSA), to provide a completely local and distributed shared frame of reference. We characterise its performance in simulation, examine the trade-offs with computational and communication cost, and to demonstrate it, we show simple but effective shape formation, and enhanced knowledge awareness within an intralogistics application. We then implement DSA on a small swarm of real DOTS robots. This paper is organised as follows, in the next section, we look at the background, Sect. 3 covers methods, Sect. 4 analyses and discusses the results, and Sect. 5 concludes.

This paper was originally presented at DARS’24. It has been extended with new material describing the implementation of DSA on a swarm of real robots and the related results.

## Background

### Gaussian belief propagation

Gaussian belief propagation (GBP) is a method of performing distributed iterative probabilistic inference or state estimation on a graph of relations between Gaussian variables by means of message passing. It is not new (Pearl, 1982; Weiss & Freeman, 1999; Bickson et al., 2008) but has received recent attention, with convergence guarantees (Su & Wu, 2015; Du et al., 2018) and applicability to distributed systems (Davison & Ortiz, 2019; Ortiz et al., 2020; Murai et al., 2024; Patwardhan et al., 2023). A widely used way to represent this inference problem is a *factor graph* (Dellaert et al., 2017); a bipartite graph consisting of nodes which are either variables or factors. Variables are quantities we wish to estimate or infer and factors, which connect to one or more variables, represent constraints on those connected variables, for example derived from some measurement of the environment.

Performing inference on a factor graph is well studied and at the heart of many robotics estimation problems. Solver libraries such as Ceres (Agarwal et al., 2023) and GTSAM (Dellaert & Contributors, 2022) are highly performant, but rely on centralised representation and processing. Davison and Ortiz (2019) make the argument that the completely decentralised and incremental processing of GBP is a better fit for future systems as processing power becomes more distributed. This naturally fits with the swarm robotics paradigm, and allows the inference of global properties by using purely local interactions.

Related consensus-based schemes achieve agreement via weighted averaging over time-varying neighbourhood graphs, Olfati-Saber et al. (2007); Jadbabaie et al. (2003). These methods update agent state directly and typically either presuppose a shared coordinate frame so state averaging is meaningful, or operate purely on a local relative basis without constructing a global frame.

**Table 1:** Notation used throughout

Symbol	Type/units	Meaning
$\mathcal {G}=(\mathcal {X},\mathcal {F},\mathcal {E})$	Factor graph	Variables $\mathcal {X}$, factors $\mathcal {F}$, edges $\mathcal {E}$
$\vec {x}_i$	$\mathbb {R}^2$	State variable (pose) in the swarm-centric frame
$b(\vec {x})$	Gaussian	Belief at *x*; by default in canonical parameters $(\eta ,\Lambda )_G$
$m_{x\rightarrow f}$, $m_{f\rightarrow x}$	Gaussian	Variable-to-factor and factor-to-variable GBP messages in $(\eta ,\Lambda )_G$
$z=(\eta ,\Lambda )_G$	Gaussian params	Factor parameters (prior or measurement) in canonical form
$h_k(\vec {x}_{k_1},\vec {x}_{k_2})$	Map	Linear relation for binary factors; here $h_k=\vec {x}_{k_2}-\vec {x}_{k_1}$
$\mu \in \mathbb {R}^2$, $\Sigma \in \mathbb {R}^{2\times 2}$	Mean, covariance	Moments parameters
$\eta \in \mathbb {R}^2$, $\Lambda \in \mathbb {R}^{2\times 2}$	Info, precision	Canonical parameters; $\Lambda =\Sigma ^{-1}$, $\eta =\Lambda \mu$
$\alpha _p$	Scalar	Precision weighting used in Eq. (4)
$r_{\textrm{damp}}\in [0,1]$	Scalar	Message damping coefficient in Eqs. (8) and (9)

Vectors are columns; Gaussians use moments form $\mathcal {N}(x;\mu ,\Sigma )$ or canonical form $\mathcal {N}^{-1}(x;\eta ,\Lambda )$ with $(\eta ,\Lambda )_G \equiv (\mu ,\Sigma )_G$

In this work, we use factor graphs; a bipartite graph $\mathcal {G}=(\mathcal {X},\mathcal {F},\mathcal {E})$ of variables $\mathcal {X}$ and factors $\mathcal {F}$ connected by edges $\mathcal {E}$. The notation used is summarised in Table 1. We use only linear 2D state representations, and only factors of one or two variables. A Gaussian variable $\vec {x}$ in the moments form $\mathcal {N}(\vec {x};\mu ,\Sigma )$ can also be represented in the canonical/information form $\mathcal {N}^{-1}(\vec {x};\eta ,\Lambda )$, connected by the identities: $\Lambda =\Sigma ^{-1},\eta =\Lambda \mu$, where $\Lambda$ is the precision matrix and $\eta$ the information vector. Hereafter, we use the compact notation $(\eta ,\Lambda )_G\equiv (\mu ,\Sigma )_G$ to express a Gaussian parameterisation; unless otherwise stated, beliefs and messages are given in canonical form. Unary factors $f_j(\vec {x}_j)$ connects to a single variable and specifies a prior, defined by the Gaussian constraint $\vec {z}_j=(\eta _j,\Lambda _j)_G$. Binary measurement factors $f_k(\vec {x}_{k1},\vec {x}_{k2})$ connects two variables $\vec {x}_{k1}, \vec {x}_{k2}$ and specifies linear relationship relation $\vec {h}_k(\vec {x}_{k1}, \vec {x}_{k2})=\vec {x}_{k2}-\vec {x}_{k1}$, with Gaussian parameters $\vec {z}_k=(\eta _k,\Lambda _k)_G$. As described in more detail in Davison and Ortiz (2019), Ortiz et al. (2021), the standard GBP algorithm requires three steps: **factor-to-variable message** $m_{f\rightarrow x}$, **variable-to-factor-message** $m_{x\rightarrow f}$, and **belief update** *b*(*x*). All messages are in the form $(\eta ,\Lambda )_G$. The schedule of operations, and nodes upon which the operations take place, can be entirely arbitrary and asynchronous, though as noted in Ortiz et al. (2021) convergence time is affected by the form of the schedule.

**Belief update:** A variable $\vec {x}_i$ has its belief updated as the product of messages from all connected factors. In canonical form, this is expressed as a sum:

1 $$\begin{aligned} b(\vec {x}_i)=\left( \sum _{f\in n(\vec {x}_i)}{\eta }_{f\rightarrow \vec {x}},\sum _{f\in n(\vec {x}_i)}{\Lambda _{f\rightarrow \vec {x}}}\right) _G \end{aligned}$$

where $n(\vec {x}_i)$ are all the factors connected to $\vec {x}_i$.

**Variable-to-factor message:** The message to a connected factor is the product of the incoming messages from all other connected factors:

2 $$\begin{aligned} m_{\vec {x}\rightarrow f_j}=\left( \sum _{f\in n(\vec {x}_i)\setminus f_j}{\eta }_{f\rightarrow \vec {x}},\sum _{f\in n(\vec {x}_i)\setminus f_j}{\Lambda _{f\rightarrow \vec {x}}}\right) _G \end{aligned}$$

**Factor-to-variable message:** For a single variable factor, this is simply the factor.

3 $$\begin{aligned} f(\vec {x}):m_{f\rightarrow \vec {x}}&= \vec {z} \end{aligned}$$

For a two variable measurement factor, this is the product of the factor and the message from the other variable, marginalising out the other variable. Alternatively, the message to a variable is the precision weighted sum of the message from the other variable and the measurement vector $\vec {z}$.

4 $$\begin{aligned} \text {Let }\alpha _{p}&= \frac{\Lambda _f}{\Lambda _f+\Lambda _p}, \bar{\alpha }_p=1-\alpha _p \end{aligned}$$

5 $$\begin{aligned} f(\vec {x}_i,\vec {x}_j):m_{f\rightarrow \vec {x}_i}&= \bigl ((\bar{\alpha }_{\vec {x}_j}\eta _f+\alpha _{\vec {x}_j}\eta _{\vec {x}_j}),\alpha _{\vec {x}_j}\Lambda _{\vec {x}_j}\bigr ) \end{aligned}$$

6 $$\begin{aligned} f(\vec {x}_i,\vec {x}_j):m_{f\rightarrow \vec {x}_j}&= \bigl ((\bar{\alpha }_{\vec {x}_i}\eta _f+\alpha _{\vec {x}_i}\eta _{\vec {x}_i}),\alpha _{\vec {x}_i}\Lambda _{\vec {x}_i}\bigr ) \end{aligned}$$

One modification we make to the original algorithm is to note that:

7 $$\begin{aligned} m_{x\rightarrow f_j} = b(\vec {x}_i) - m_{f_j\rightarrow x} \end{aligned}$$

Variable nodes just calculate their beliefs and send them as messages, and factor nodes locally calculate what the variable-to-factor message would have been by subtracting the last message sent to that variable. This minimises the non-local knowledge needed in any node and more fairly distributes computation around very connected nodes.

**Message damping** As noted in Su and Wu (2015), message damping often improves convergence. We only damp messages from factors to variables, such that the message components $(\eta ,\Lambda )$ are replaced:

8 $$\begin{aligned} \eta '_{t+1}&= (1-r_{\textrm{damp}})\eta _{t+1} + r_{\textrm{damp}}\eta _{t} \end{aligned}$$

9 $$\begin{aligned} \Lambda '_{t+1}&= (1-r_{\textrm{damp}})\Lambda _{t+1} + r_{\textrm{damp}}\Lambda _{t} \end{aligned}$$

Throughout this work, we use a damping factor of $r_{\textrm{damp}}=0.8$, arrived at empirically during initial experimentation as a compromise between slow convergence and a tendency to oscillation.

### Shape formation

Shape or pattern formation in robot swarms is widely studied. The use of a swarm to perform a task is often implicitly or explicitly dependent upon that swarm maintaining a particular shape or covering a particular area. Often, a necessary part of forming a shape is the use of simpler behaviours such as dispersion, aggregation, alignment. Notably Reynolds (1987) introduced simple rules to produce complex flocking behaviour. Potential fields or virtual springs are a common approach (Pan et al., 2019; Sabattini et al., 2011) but often use assumptions such as knowledge of position. Rubenstein et al. (2014) used kilobots (Rubenstein et al., 2012) to build arbitrary 1-connected shapes by individual robots extending a seed formation and localising themselves against already positioned static robots, likewise Li et al. (2019) progressively construct a shape with new agents positioning themselves against those already present. Also there are bioinspired approaches such as morphogenesis (Carrillo-Zapata et al., 2019; Slavkov et al., 2018). See Oh et al. (2017) for a recent review.

Several systems have demonstrated shape formation on real robot swarms; with kilobots, Rubenstein et al. (2014), constructing a common reference frame as robot accumulate around four seed robots. Li et al. (2019) validate their method on Khepera IV robots (Soares et al., 2016), synthesising relative position information from a global tracking system. Stolfi and Danoy (2024) use E-puck2 robots with synthesised range-and-bearing sensing of other robots over distances much larger than the shape being formed. Montijano et al. (2016) use consensus to reach an agreed formation with three quadrotors. Relative positioning is inferred using cameras to observe visible features on floor or ceiling and homography, with scale agreed by consensus. Except for the Kilobot work, these systems typically operate directly in relative coordinates and do not construct an explicit shared global frame. In contrast, we treat the same local relative measurements (and odometry) as factors in a distributed inference problem, and shape formation is layered on top of this frame.

### Swarm logistics

Swarm logistics can be regarded as a real-world application of foraging (Winfield, 2009; Liu & Winfield, 2010; Pitonakova et al., 2018; Talamali et al., 2020). Robots must leave a *nest* area and seek resources to be returned to the nest. The analogy is obvious. As a use case, we focus on robot swarms used for intralogistics. As noted above, we specifically focus on the out-of-the-box, easily deployable solutions for everyday environments, as described in Jones et al. (2020). Even simple random walk algorithms are capable of effective retrieval in logistics applications, (Milner et al., 2022a, b). Messages from users propagate from robot to robot, robots store and retrieve items, all in parallel and without central resources. Although simple, this scenario encapsulates many issues that will need to be solved for viable swarm logistics systems to become a reality.

## Methods

We work in simulation and the real world. As such, for simulation we use an abstract model of our real robots, the DOTS (Jones et al., 2022); each robot is modelled as a 2 kg disk, 250 mm in diameter, that can move holonomically at up to $$v_{\textrm{max}}=\, 1\hbox{ms}^{-1}$$, and can sense and identify other robots and objects to a distance of $$r_{\textrm{sense}}=$$ 0.5 m in simulation, 1 m for the real robots. Communication is possible between any robots that can sense each other. In simulation, each robot has imperfect sensors distorted by Gaussian noise; a velocity sensor $$\vec {v}_{\textrm{sense}}=GT(v_{\textrm{robot}}) \cdot \mathcal {N}(1,\sigma ^2_{\textrm{velocity}})$$, and a relative position sensor $$\vec {p}_{\textrm{object}}$$$$GT(\textrm{object})-GT(\textrm{robot})$$$$+\mathcal {N}(0,\sigma ^2_{\textrm{position}})$$ where *GT*(*k*) is the ground truth from the simulator. The robot maintains odometry $$\vec {p}_{\textrm{odom}} = \sum \vec {v}_{\textrm{sense}}\Delta t$$, $$\Delta t=\frac{1}{60}$$s, integrated velocity since the last variable node was created.

### Simulator

The simulator is written in C++, using the Box2D (Catto, 2009) physics engine. Updates to the simulation occur at 60 Hz. The arena is a square area with fixed walls. Robot motion is modelled as a disk with friction sliding on the arena surface, collisions between robots and with walls follow physics. Low level proportional control of the force applied to the robot body is used to satisfy the commanded velocity. As well as robots, there can be objects that can be detected by the robots. For each robot a list of robots and objects within $r_{\textrm{sense}}$ range is maintained, and at each timestep a set of abstract senses is constructed and a controller routine is executed on those senses to generate a commanded velocity. The simulated senses and actuator are shown in Table 2.

**Table 2:** Robot senses and actuator

Senses	Description
$\vec {v}_{\textrm{sense}}$	Velocity
$\vec {p}_{\textrm{odom}}$	Odometry since last variable node
$\vec {p}_{\textrm{robot}}=\vec {x}_i+\vec {p}_{\textrm{odom}}$	Inferred position of robot relative to swarm centroid
$\{\vec {p}^1_{\textrm{object}},..,\vec {p}^n_{\textrm{object}}\}$	List of other robots and objects within $r_{\textrm{sense}}$ radius
$\angle _{\textrm{neighbours}}$	Direction of nearest neighbour robots
$\text {converged}$	True when elapsed time $>t_{\textrm{convproxy}}$
Actuator	
$\vec {v}_{\mathrm {cmd\_vel}}$	Commanded velocity of robot

**Fig. 1:**
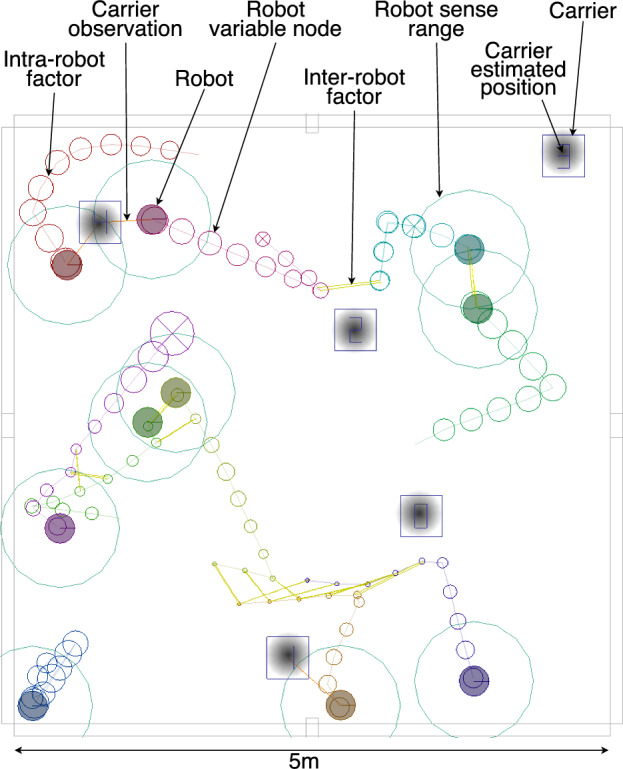
Simulation of robots performing DSA-RW to locate cargo carriers, with various elements of the visualisation labelled. Within the carrier squares are overlayed the current swarm estimates of the carrier position, already showing good correspondence after two minutes of simulated time

The baseline controller performs a random walk moving at $v_{\textrm{fast}}=0.5\,\hbox{ms}^{-1}$ in a random direction $t_{\textrm{rwduration}}=\max (0.1,\mathcal {N}(2,1))$ seconds, before choosing a new direction and duration. Collisions are treated as ballistic. This behaviour is denoted DSA-RW (Distributed Spatial Awareness - Random Walk) (Fig. 1).

### Distributed spatial awareness

We construct a shared reference frame for the swarm in the following way: Each robot builds a local factor graph with variables representing 2D pose, unary anchor factors connecting to a single variable, and binary relative measurement factors connecting two variables. Each robot shares synchronised time, and at regular intervals $t_{\textrm{node}}$ from a starting epoch a new timestep $ts_i$ is issued and a new variable node $\vec {x}_i$ is created. At any given time, the pose of the robot relative to the shared reference frame is $\vec {p}_{\textrm{robot}} = \vec {x}_i + \vec {p}_{\textrm{odom}}$.

The first node to be created will have an anchor factor connected to it with a weak prior of pose (0, 0). Successive nodes have a measurement factor connecting them: $f_{\vec {x}_i\rightarrow \vec {x}_{i-1}}(\mu =\vec {p}_{\textrm{odom}},\Sigma =\sigma ^2_{\textrm{velocity}}I_2)$. As new variable nodes are created, old ones are removed to maintain a maximum number of nodes $n_{\textrm{window}}$, with the now oldest variable having an anchor factor attached to it set to the belief of that variable. At every $t_{\textrm{message}}$ interval, a factor node is chosen at random, and messages are propagated to each connected variable according to the GBP algorithm. Each connected variable then has its belief updated.

As described, this local factor graph is purely a bounded time window sampling of the odometry of the robot. In order to connect the local factor graphs together into a swarm whole, we add two things: 1) When a new variable node is created, robots observe other robots within their sensory range and create measurement factors $f(\mu =\vec {p}_{\textrm{object}},\Sigma =\sigma ^2_{\textrm{position}}I_2 )$ that link the new variable node on the observing robot to the observed robot. These factors, termed outward-facing, are given the current timestep $ts_i$ and the ID of the other robot. This uniquely identifies the variable at the other robot that it links to. 2) Robots in sensory range exchange GBP messages. At the same $t_{\textrm{message}}$ interval as above, the robot randomly chooses a single other robot from any within $r_{\textrm{sense}}$ range and sends a message request. This consists of a list of the timesteps of all outward-facing factors that connect to the other robot. The responding robot replies with a list of beliefs from the variables those factors uniquely connect to, and a list of messages from remote factors that connect to local variables of the requesting robot.

**Fig. 2:**
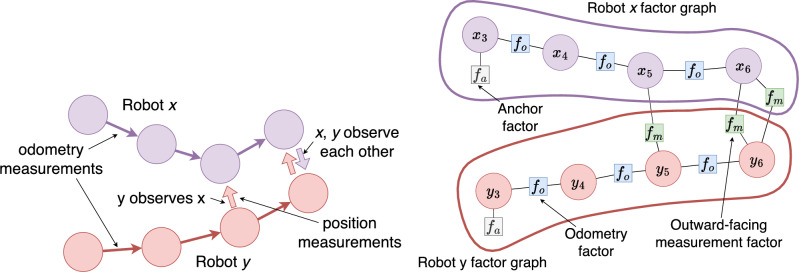
Robots creating connected factor graphs. **Left:** Two robots move on trajectories, making odometry measurements, that bring them within sensing range of each other. **Right:** The internal factor graphs that are created on each robot, assuming $n_{\textrm{window}}=4$ and some time has passed. The oldest variable node in each graph, $x_3,y_3$, has an anchor factor. Between each variable node is an odometry measurement factor node. In timestep $ts_5$, robot *y* has observed robot *x*, creating an outward-facing relative position measurement factor. In $ts_6$, both robots have observed each other

The factors linking variable nodes on different robots create the constraints that produce convergence of local pose estimates into a shared consistent state, i.e a shared reference frame. This process is illustrated in Fig. 2.

Assume that robot *x* has sensed robot *y*, this is the process that follows. It sends a list of the timesteps of outward-facing factors connected to robot *y*, which will just be $\{6\}$, for the factor connecting $x_6$ to $y_6$. Robot *y* replies with $\{b(y_6), m_{f\rightarrow x_6}, m_{f\rightarrow x_5}\}$. Robot *x* has now got message information needed to update the beliefs of $x_5$, $x_6$, and to create an outward message from $f_m$ attached to $x_6$.

#### Convergence

**Fig. 3:**
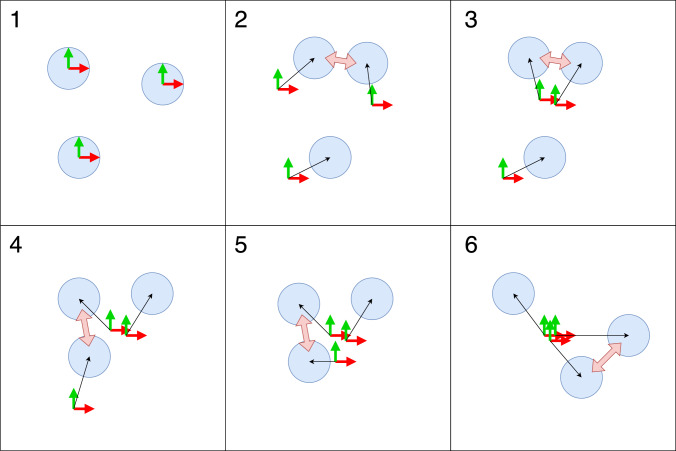
Illustration of shared reference frame convergence. 1) All robots start by thinking they are at the centre of the swarm. 2) Observation and communication imposes constraints on location of the swarm centroid; the top two robots communicate.. 3) .. and each robot updates its own estimate. 4) More communication imposes further constraints. 5) Origin estimates move closer.. 6) ..and approach convergence

As robots encounter each other and exchange messages, and individual robots perform message passing on their individual fragments of the factor graph, the pose of each robot becomes constrained against others, thus the assumed origin or reference frame of each robot converges, Fig. 3. A necessary condition for convergence is that all parts of the graph must have, at some point, been connected. For *n* robots, we expect this to happen after $n_{\textrm{enc}}=\frac{1}{2}n\ln n$ new robot pair encounters, Erdős et al. (1960).

It is important to note that all factor messages convey relative constraints, and all variable belief messages convey local probabilistic historical pose estimates, over a stochastically connected joint factor graph. This process performs inference to solve for a consistent swarm-centric frame, with no prior alignment. It is dynamic, and will never completely converge, since robots are moving and have noisy perceptions, and the whole graph does not remain fully connected.

The error is the mean deviation from the swarm centroid of the robot origins:

10 $$\begin{aligned} r_{\textrm{error}}=\frac{1}{n_{\textrm{robots}}}\sum _{i=1}^{n_{\textrm{robots}}}|\vec {r}^i_{\textrm{origin}}-\mu _{\textrm{origin}}| \end{aligned}$$

This is only knowable from the global perspective of the simulator. We define the convergence time $t_{\textrm{conv}}$ as the time taken for error to reach $r_{\textrm{error}}<2\sigma _{\textrm{position}}$ since position observation noise dominates convergence error.

To make the shared reference frame useful to a swarm system, individual agents within the swarm need to know when they can rely on estimates but they have no access to the global measure $r_{\textrm{error}}$. In characterising the system, we collect data on the time taken for agents to encounter at least half the robots in the swarm, denoted $t_{\mathrm {met\_half}}$. We reason that this, with some constant, should be a reasonable proxy for the time taken to build a fully converged reference frame:

11 $$\begin{aligned} t_{\textrm{convproxy}} = \beta \cdot t_{\mathrm {met\_half}} \end{aligned}$$

To implement this, each robot keeps a count of the number of unique other robots it has encountered. When this exceeds half the size of the swarm, the time is noted and $t_{\textrm{convproxy}}$ calculated. Only when the elapsed time is greater than this is it possible to use the shared reference frame derived $\vec {p_{\textrm{robot}}}$. The two example algorithms below wait for this before switching behaviour from the baseline random walk controller DSA-RW.

### Application: shape formation

Because each robot has access to the shared reference frame, it is easy to construct algorithms for swarm-wide shape formation. To demonstrate this, we use a simple algorithm called DSA-SF (Distributed Spatial Awareness - Shape Formation) where the shape is defined functionally, $f_{\mathrm {in\_shape}}(p_{\textrm{robot}})$, and shown in Algorithm 1. Each robot has two modes of behaviour; when not inside the shape according to its estimated position $\vec {p}_{\textrm{robot}}$, it uses the baseline behaviour DSA-RW. When inside the shape, it slows down to $v_{\textrm{slow}}=0.1\cdot v_{\textrm{fast}}$, and if there are any neighbours, it moves towards them, causing classic swarm aggregation.

**Algorithm 1:**
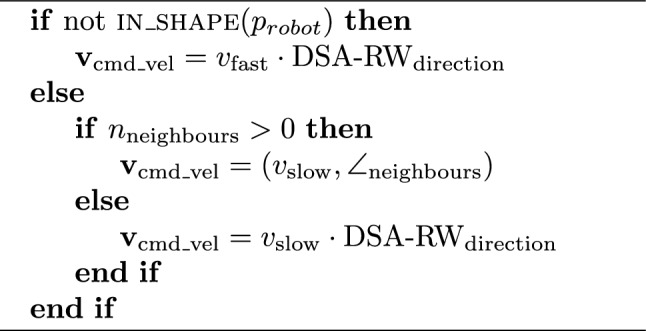
DSA-SF Shape formation

We define some simple shapes such as a circle, vertical and horizontal lines, and wavy lines, and switch between them at intervals.

### Application: intralogistics

As noted above, even random walkers are capable of performing simple swarm logistics operations. We want to enhance the performance of such systems using the shared reference frame. A key component to a logistics system is knowledge of the location of cargo carriers. Carriers *C* are dynamic objects that may move. Each robot maintains a set of Gaussian variables and an associated time of observation $c(i)\equiv (\vec {x}_{\textrm{carrier}}^{i},t_{\textrm{observed}}^i), i \in C$, one for each possible carrier. When a robot observes a carrier *k*, it sets the tuple $c(k)=((\vec {p}_{\textrm{robot}}+\vec {p}_{\textrm{carrier}},\sigma ^2_{\textrm{position}}+\sigma ^2_{\textrm{robot}})_G,t)$. Each time a robot exchanges messages with another robot, it sends a list of the observation times $t_{\textrm{observed}}^i,i\in C$ it has, including $t=0$ for carriers for which it has no observation. The other robot replies with any carrier observations it has that are more recent, these are used to replace the older observations. More recent observations are privileged, even if they may have greater positional uncertainty, since the carrier may have moved.

**Algorithm 2:**
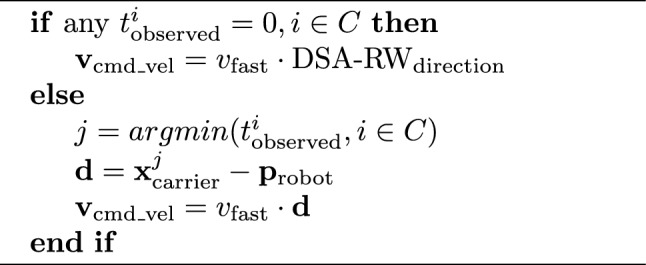
DSA-KE Knowledge Enhancement

The way the robots move has an impact on the acquisition of knowledge about the environment. When the swarm has no knowledge, the goal is to cover as much of the arena area as possible. For this, we use the baseline behaviour DSA-RW. Once the swarm has a certain level of awareness, it becomes possible to use this information to guide swarm exploration so as to maximise knowledge. This behaviour is called DSA-KE (Distributed Spatial Awareness - Knowledge Enhancement), and is identical to DSA-RW until a robot has some information about all the carriers in the environment. At that point, instead of choosing a direction at random, a robot will choose the direction of the carrier which it has oldest knowledge about, thus the swarm as a whole seeks to minimise overall uncertainty, Algorithm 2.

In order to test the quality of knowledge acquisition in a dynamic environment, we make the carriers move position. This is specified with a single parameter, the aggregate mean carrier velocity $v_{\mathrm {c\_agg}}$. Given $n_{\textrm{carrier}}$ carriers, a carrier is selected at random and moved a fixed distance of 1 m into a random location at a velocity of $v_{\mathrm {c\_agg}}\cdot n_{\textrm{carrier}}$. The mean perception error of the swarm is given by:

12 $$\begin{aligned} s_{\textrm{error}}&= \frac{1}{n_{\textrm{robots}}n_{\textrm{carrier}}}\\ &\quad\sum _{j=1}^{n_{\textrm{robots}}}\sum _{i=1}^{n_{\textrm{carrier}}}|c(i)_{\textrm{est}}^j-c(i)_{\textrm{gt}}| \end{aligned}$$

where $c(i)_{\textrm{gt}}$ is the ground truth position of a carrier, transformed into the swarm frame.

### Application: DOTS robots

Bringing techniques from simulation into the real world is an important step toward applications. Here we detail how we implement DSA on the DOTS robots. The robots run within a custom-built arena of 3.7m $\times$ 3.7 m. Each has a controlling RockPi 4 single board computer (SBC) running ROS2, and is connected to a dedicated WiFi access point. A PC server is used for data collection.

In our simulation model, robots can sense the relative position of all other nearby objects within a fixed range with added Gaussian noise; an idealised sense. How should we construct relative object sensing in our real robots?

In reality, each robot has four wide-angle cameras around their periphery, with approximately $90^{\circ }$ horizontal field of view. The two front cameras are closer together, with overlapping views. Cameras are not calibrated individually; during construction of the robots, the intrinsics and distortion parameters were measured and averaged for a subset of cameras. Because visual processing for arbitrary object recognition is expensive and the controlling RockPi 4 single board computer (SBC) is relatively low power, we rely on fiducial tags in the environment to facilitate comprehension of the world. We reason that future deployment will have access to much higher computation ability, making this a reasonable analog for full object recognition. Each camera is connected to a Raspberry Pi Zero, which runs fiducial detection on-board (Jones & Hauert, 2023), making a stream of tag detections from each camera available to the SBC.

#### Sensing other robots

**Fig. 4:**
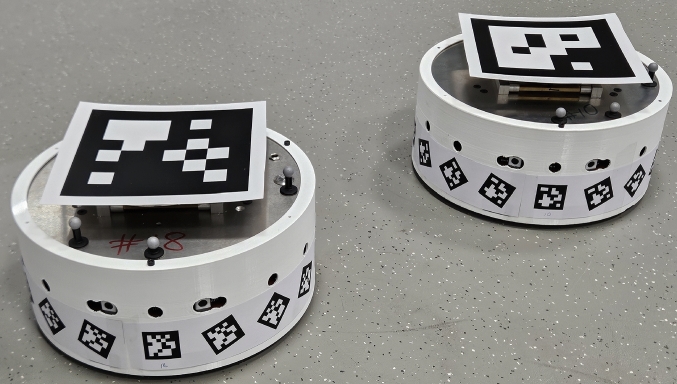
Two DOTS robots, with identifying fiducial tags around their bodies. Also visible are the two front-facing cameras and large top mounted fiducial tags for ground truth tracking

In this application, we use a band of fiducial markers around the body of the robots to allow them to sense each other visually. Each robot is allocated a unique tag ID. This tag is replicated 16 times around the robot, see Fig. 4, with the *i*th tag rotated by $i2\pi /16$ about its *z*-axis.^1^ This band of ID tags have known and fixed locations relative to the robot. Because we know that both the observing and observed robots are on the same plane, by calculating the *z*-angle of observed markers, we can disambiguate which of the 16 tags are being observed and thus know the physical location of the marker corners in the frame of the observed robot. Using the OpenCV solvePnP function with the camera intrinsics and distortion parameters, we get the transform $^\textrm{cam}T_{\textrm{idband}}$. Combining with with observing camera extrinsics $^\textrm{robot}T_{\textrm{cam}}$ we get the relative poses of the two robots $^\textrm{robot}T_{\textrm{idband}}$. The full relative pose in SE(2) is not necessary for this application but we intend to use it in future work. Initial experiments showed that achieving reliable fiducial detection between relatively moving robots was difficult. Fiducial detection relies on sharp image edges, but relative movement between robots results in lateral movement of fiducials across a camera view, inducing image blur, increasing with reduced robot-robot distance. This limits both the velocity of the robots and the close range detection distance. Reducing robot velocity $v_{\textrm{robot}}$ to $0.25 \,\hbox{m s}^{-1}$ (from $0.5\,\hbox{m s}^{-1}$ in simulation) and extending the allowed sensing range $r_{\textrm{sense}}$ to 1 m (from 0.5 m in simulation) resulted in a reasonable detection rate.

The odometry noise model of metres per metre travelled still approximates the real robots. But for relative position measurement using cameras, the application of a fixed uncertainty to the relative (*x*, *y*) position is not realistic; position measurement is effectively range and bearing, which is non-linear. Pixel measurement noise *P* maps predominately to range *r*; at $r=1 \hbox {m}, \frac{dr}{dP}\approx -0.11\,\hbox{m}$ whereas angle $\frac{d\theta }{dP}\approx 0.18^{\circ }$, or about 3.2 mm displacement. To handle this properly, we would need to use non-linear factors, and perform linearisation during inference, but to keep the same code as used in simulation, we opt to simply add a larger uncertainty, scaled to distance: $\sigma _{\textrm{position}} = 0.02 + 0.04r$.

#### Implementation in ROS2

All robots run on a common ROS2 system, and they all have synchronised clocks. Each robot receives a signal on the /launch topic and this is used to trigger the ROS node handling the DSA processing to start. These nodes on different robots run in lockstep with skew between them of less than 50 ms. The simulation code relies on unique identifiers being created for each factor graph node based on the robot ID and the current timestep $ts_i$, so these can be referenced during message exchange. This scheme is unchanged except to tie the robot ID to the physical robot name, rather than simulation object creation order. Update period for the factor graph was fixed at $t_{\textrm{message}}=0.1 s$. As with the simulation, a list of robots within $r_{\textrm{sense}}$ range that have been detected is maintained, the relevant transform being broadcast to the local tf tree. At each update, if there are any robots within range, as determined by whether they are visually identified and measured as less than 1 m away, one is picked randomly and the message exchange protocol described in Sect. 3.2 executed. This is implemented as a ROS2 service request. The response is not guaranteed, and is not synchronous, but this does not matter for the GBP algorithm; returning data if it arrives is placed in the inward message slots of relevant factor and variable nodes.

Robot odometry from wheel velocity $\vec {p}_{\textrm{odom}}$ together with the latest variable node $\vec {x}_i$ is used to maintain the estimated position relative to the swarm $\vec {p}_{\textrm{robot}}$. This is also broadcast to the local tf tree.

In order to maintain the requirements of the DSA code that robot pose is pure 2D, we use a synthetic compass sense and low level proportional controller acting only on angular velocity to maintain a constant robot heading. All robots run localisation on-board, using odometry, IMU, and observation of twelve large fiducial markers in fixed known locations on the arena walls to establish their global pose within the arena. The compass sense is synthesised from this pose orientation; there is no access by DSA to the position information, it is not used within this work.

#### Pattern formation

We perform experiments with six DOTS robots. As well as their peripheral bands of identifying fiducials, each robot is fitted with a larger unique fiducial marker on its top surface for ground truth tracking. At the start of a run, the robots are placed in random locations within the arena and the DSA estimation is reset. Each run consists three repetitions of Algorithm 3; a mixing period of $t_{mix}=30 s$ consisting of a random walk at a velocity of $v_{\textrm{robot}}=0.25\,\hbox{ms}^{-1}$. Once the mixing period is complete, the robots try and form a hexagon of radius 0.7 m around the origin of the shared reference frame. Each robot has a fixed target vertex in the hexagon pattern, with a simple proportional controller driving robot linear velocity. At all times collision avoidance is active, with obstacle detection via 16 laser Time-of-Flight sensors. Any obstacle less than 0.2 m away, causes a random direction roughly opposite the obstacle to be chosen.

Once the hexagon pattern has stabilised, two more sets of mixing period and hexagon formation were initiated, with the DSA estimation retaining accumulated state. Five runs in total were performed. For each run, we collect video recordings, and ROS2 bag files of relevant topics, for later analysis.

**Algorithm 3:**
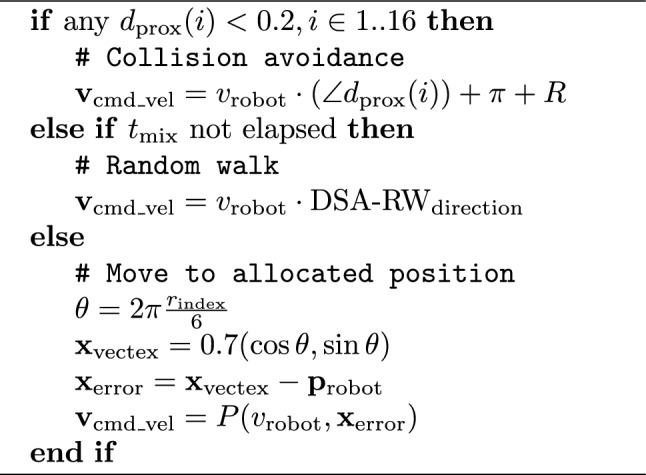
DOTS demonstration

## Results

We measured the performance of various metrics of interest. In each case, simulations were run 50 times with different random seeds for each datapoint. Standard parameters are shown in Table 3. DOTS robot experiments recorded video and bag file data which was post-processed to get unified datasets.

**Table 3:** DSA parameters

Parameter	Value	Description
$n_{\textrm{window}}$	10	Max number of variable nodes in local factor graph
$t_{\textrm{node}}$	0.5 s	New variable node creation interval
$d_{\textrm{robot}}$	0.25 m	Robot diameter
$r_{\textrm{sense}}$	0.5 m	Object and robot sense and communication radius
$\sigma _{\textrm{velocity}}$	0.1 m/m	Velocity sense noise (metres per metre travelled)
$\sigma _{\textrm{position}}$	0.02 m	Position sense noise
$v_{\textrm{fast}}$	${0.5}\,\hbox{ms}^{-1}$	Movement performing DSA-RW and DSA-KE
$v_{\textrm{slow}}$	$0.05\,\hbox{ms}^{-1}$	Movement within shape while performing DSA-SF
*DOTS parameters where different*		
$r_{\textrm{sense}}$	1.0 m	Object and robot sense and communication radius
$\sigma _{\textrm{position}}$	$0.02+0.04r$ m	Position sense noise, *r* is distance to robot
$v_{\textrm{robot}}$	$0.25\,\hbox{ ms}^{-1}$	Robot movement speed

### Convergence, computation, and bandwidth

We examine how long the distributed factor graph takes to converge, $t_{\textrm{conv}}$, and how much computation and bandwidth is used in different scenarios. An example video of 10 robots reaching convergence is available at https://youtu.be/3S9Ko356eiY.

**Fig. 5:**
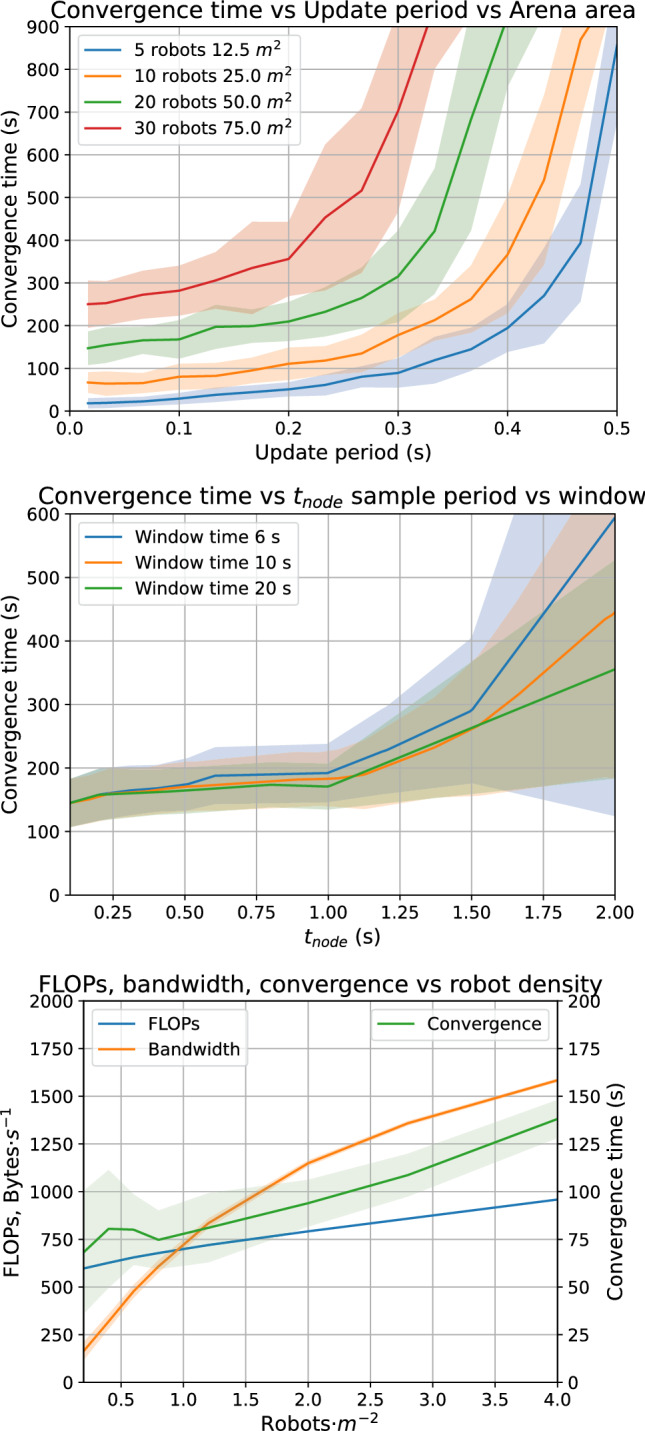
**Top:** Convergence time with different factor graph update rates and different arena areas, with fixed robot density. Reduction in convergence time is minimal below 0.1 s update period. **Middle:** Convergence time with different $t_{\textrm{node}}$ periods, at three different window lengths, using 20 robots in a $50\,\hbox{m}^{2}$ arena. **Bottom:** For a fixed update period of 0.1 s and fixed arena area of $25\,\hbox{m}^2$, computation, convergence time, and particularly bandwidth are dependent on robot density

Firstly, we look at different $t_{\textrm{message}}$ update periods of the factor graph, and different numbers of robots in an arena sized to keep a constant robot density of $0.4\,\hbox{ ms}^{-2}$. See Fig. 5 top. Convergence takes longer when there are robots over a larger area, this is expected, since all robots have to be able to influence each other, though not necessarily by direct communication, that is, the factor graph over time must be connected. Increasing density with constant area has a small linear effect on convergence (bottom) due to the larger total size of the graph, but the dominating factors are update rate and arena area. There is little gain from rapid updates over 10 Hz.

Because convergence requires the factor graph be connected, we next examine how $t_{\textrm{node}}$ controls the rate of new edge formation. The number of nodes $n_{\textrm{window}}$ is less informative than the total history length, so the middle graph represents interpolated slices through three different fixed history windows. This shows a clear knee in convergence time $t_{\textrm{conv}}$ at $t_{\textrm{node}}=1\,\hbox{s}$. We explain this with reference to the time that meeting robots remain within sense range $\tau _{\textrm{in}}\approx 1\,\hbox{s}$. For $t_{\textrm{node}} <\tau _{\textrm{in}}$, we are effectively continuously sampling the environment, so we record new edges at a rate independent of $t_{\textrm{node}}$, determined only by the physics of encounters ($n_{\textrm{robots}},v_{\textrm{robot}},r_{\textrm{sense}}$, and arena area), which is constant here. For $t_{\textrm{node}}>\tau _{\textrm{in}}$, sampling, not physics, limits progress; more robot encounters start to take place between the sample points, so the time to reach connectivity, and thus convergence, grows roughly linearly with $t_{\textrm{node}}$. Smaller history lengths have worse convergence, there is more ‘forgetting’, and fewer constraints for the GBP algorithm to work with; a six second window with $t_{\textrm{node}}=2\,\hbox{s}$ only has three nodes.

Computation and bandwidth are both proportional to update frequency so we use a fixed sized arena of $25\,\hbox{m}^2$ and update rate of 10 Hz to look at other factors. All operations are performed in 32 bit floating point. Since we are working in 2D space with linear factors, the precision matrix $\Lambda$ can be represented as a single number. Belief update needs 3 operations per attached factor (Eq. 1). Factor message generation only needs computation for measurement factors; $2\,\text{(Eqn}$4) $+1+2+2+1\,\text{(Eqn}$ 5)$+2+2+1\,\text{(Eqn}$6) $=13$ operations. Assuming a naive message protocol, every request has an overhead of 12 bytes, and 4 bytes per outward-facing factor, and each response has an overhead of 12 bytes, and 16 bytes per returning belief and factor-to-variable message.

There is a weak dependence of computation on density, as there are more chances to create additional outward-facing factors. Bandwidth is strongly dependent on robot density, as there are many more opportunities for a robot to communicate. It should be noted that the raw figures for supporting the shared reference frame are remarkably low; for a $25\,\hbox{m}^2$ arena, the swarm will converge in less than 60 s, with each robot using only a few hundred floating point operations and exchanged message bytes per second. This is achievable even on low cost processors.

**Fig. 6:**
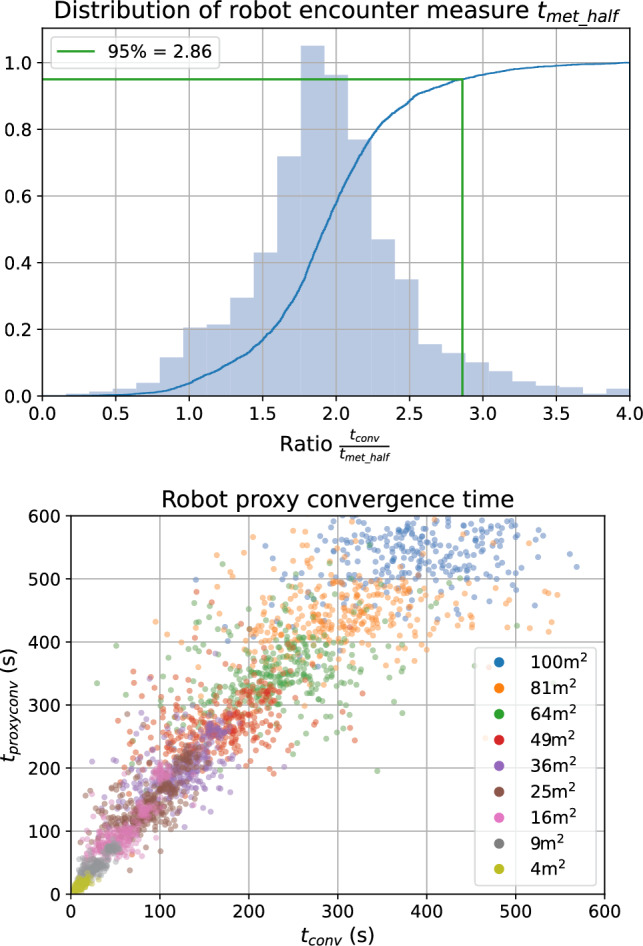
The distribution of the robot encounter measure $t_{\mathrm {met\_half}}$ over 2886 simulations with different numbers of robots between 5 and 100, and different arena sizes between $4\,\hbox{m}^2$ and $100\hbox {m }^2$. Using a constant $\beta =3$ ensures the proxy for convergence time $t_{\textrm{convproxy}}$ exceeds the true convergence time $t_{\textrm{conv}}$ in more than 95% of simulation. Bottom scatter plot coloured according to arena area

In order to determine an appropriate value for $\beta$ (Eq. 11), we ran a set simulations over different numbers of robots between 5 and 100, and different arena sizes between $4\,\hbox{m}^2$ and $100\,\hbox{m}^2$, fixing the update period $t_{\textrm{message}}=0.1$ s. Simulations were run for 1000 simulated seconds. Out of 4200 simulations, 2886 were able to find an initial placement for the robots in the arena size, and reached convergence within the simulation run time. We can see from the graphs in Fig. 6 that using a value of $\beta =3$ ensures the proxy measure $t_{\textrm{convproxy}}$ exceeds the true measure $t_{\textrm{conv}}$ in 95% of simulations.

### Shape formation

**Fig. 7:**
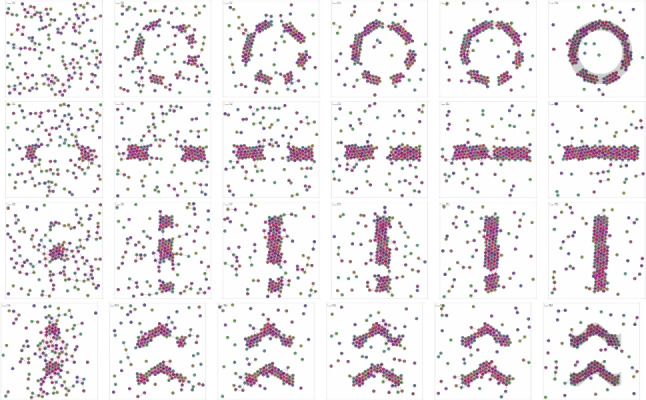
Shape formation. 150 robots perform random walk DSA-RW when outside shape, and slow down and aggregate when inside shape, taking about 40 s to form each pattern

We ran simulations using 150 robots, with an arena size of 7.5 m per side. After reaching convergence, each robot followed Algorithm 1, with the shape function switching at regular intervals. Figure 7 shows snapshots of the process, with the desired shapes emerging within about 40 s in each case. The video at https://youtu.be/ps5Wf-3UHr0 shows this process in full.

The algorithm is extremely simple, yet forms and cleanly transitions between shapes even without fine tuning of parameters.

### Intralogistics

**Fig. 8:**
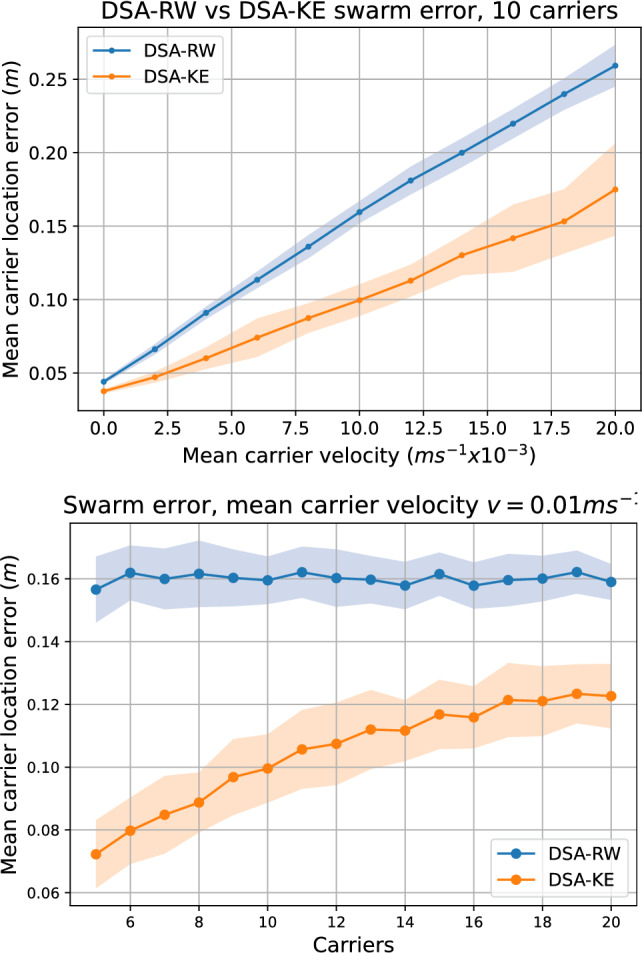
Swarm carrier location error $s_{\textrm{error}}$ with the two robot behaviours while varying the carrier velocity (top) and the number of carriers in the arena (bottom). The number of robots is fixed at 10. Shaded areas indicate $\pm \sigma$ over 50 different random seeds. As the mean carrier velocity increases, so does the error, which is expected. With a fixed mean carrier velocity and sweeping the number of carriers, we see roughly constant error with the random walk of DSA-RW, but much lower error with DSA-KE as it actively seeks to enhance knowledge, particularly with low carrier numbers. In all cases, the DSA-KE behaviour results in much lower swarm perception error

Figure 8 shows how the two movement behaviours DSA-RW and DSA-KE perform in acquiring knowledge about the dynamic carrier environment. At zero carrier velocities, the swarm quickly reaches low $s_{\textrm{error}}$ values, around 0.04 m. Since the position sense has injected noise of $\sigma _{\textrm{psense}}=0.02\,\hbox{m}$, this is a reasonable lower bound. As the velocity of the carrier movement increases, the error goes up as unobserved carriers can move further from their last know positions.

The effectiveness of DSA-KE in improving carrier position estimation in the swarm by actively moving to areas where knowledge is weaker, is clearly apparent. In all cases over different numbers of carriers and different carrier velocities, we see improvement, 38% with 10 carriers and mean velocity $0.01\,\hbox{ms}^{-1}$. When looking at performance vs the number of carriers, we see that DSA-RW is roughly constant, whereas DSA-KE performs extremely well with lower numbers of carriers. Clearly visible in the video available at https://youtu.be/r53Z1O1pfxk is a rather interesting emergent flocking behaviour; as one robot heads towards the carrier it knows least about, the backwards propagation of information from the leading robot on average causes trailing robots to be in a better position to seek the next low-knowledge carrier, cohering the swarm. We illustrate the dynamics of this in more detail with the video at https://youtu.be/5Ad-HzJYnM8, colour coding the least recently visited carrier, and colouring robots with their current target.

The good relative performance of DSA-KE falls off at higher numbers of carriers, though it is still 24% better than DSA-RW with 20 carriers. It does suggest that a better strategy for high carrier numbers would ensure a greater degree of dispersion.

### DOTS robots

**Fig. 9:**
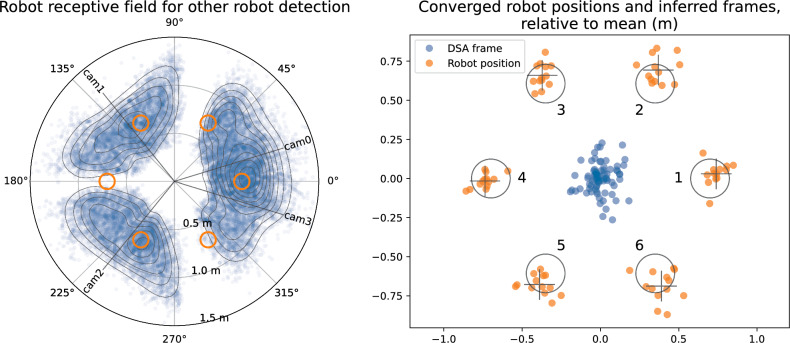
**Left:** The visual receptive field of the DOTS robots. All visual detections by robots of other robots while performing random walk are shown, totalling 38,663, with density contours. Also shown are the angles of the four cameras, and orange circles at the locations where other robots would need to be detected for optimal hexagon shape formation. **Right:** Locations of robots and each of their inferred frames for all converged pattern formation attempts. Circles show ideal robot positions and robot index, crosses mark mean actual locations

Firstly, we characterise the performance of the visual recognition system. Figure 9 left shows all the robot detections of other robots while the swarm was performing a random walk, a total of 38,663 datapoints. These are plotted as a polar graph, and show that the receptive field is not perfect, with significant blind spots at about $75^{\circ }$ and $180^{\circ }$ from the robot forward direction. Most detections were in the distance range 0.5–1.0 m. Overlaid on the graph of the robot receptive field are six orange circles, which illustrate where neighbouring robots will appear when in the ideal hexagon pattern. Two of the six robots in the pattern have little to no visibility of their neighbours; to the rear and to the front left or right.

Range and bearing error are shown in Table 4. Bias and spread of error for range was quite low, given the value of $\frac{dr}{dP}=-0.11\,\hbox{m}$, demonstrating effective subpixel localisation of fiducial corners. Bearing error was much higher than could be explained by pixel error, and more likely due to inaccuracies in keeping robots pointing to a fixed heading.

**Table 4:** Range and bearing measurement error

Measurement	Mean error	$\sigma$ error
Range (m)	0.012	0.044
Bearing ($\angle ^{\circ }$)	− 1.2	3.4

**Table 5:** Mean deviation $r_{\textrm{error}}$ of robot DSA frames

Run	Pattern 1	Pattern 2	Pattern 3
1	0.102	0.078	0.067
2	0.138	0.181	0.094
3	**0**.**328**	**0**.**344**	0.168
4	0.062	0.058	0.146
5	0.123	0.050	0.062

Bolded numbers show pattern formation where convergence was not successful

**Table 6:** Mean vertex positions, vertex error $v_{\textrm{error}}$, and effective pattern radius in metres (excluding Run 3 Patterns 1,2)

Robot	$\bar{x}$	$\bar{y}$	$v_{error}$	*r*
Robot 1	0.740	0.030	0.069	0.741
Robot 2	0.370	0.694	**0**.**102**	0.786
Robot 3	− 0.372	0.659	0.071	0.757
Robot 4	− 0.738	− 0.016	0.060	0.738
Robot 5	− 0.389	− 0.678	0.088	0.782
Robot 6	0.389	− 0.687	**0**.**115**	0.790
			0.084	0.766

Note that robot 2 and robot 6 have worse $v_{\textrm{error}}$, as would be expected from the visual receptive field

**Fig. 10:**
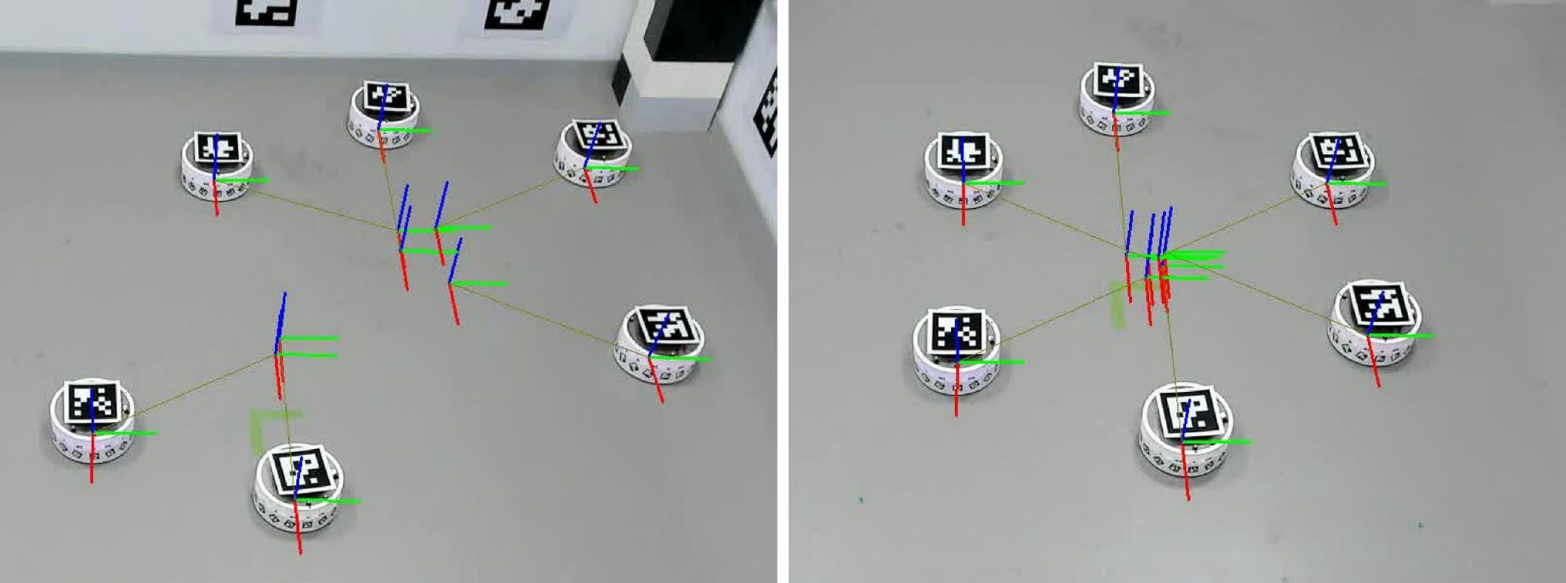
Worst and best $r_{\textrm{error}}$ of robot DSA reference frames. From video with overlaid annotation of robot ground truth and DSA frames. Left is Run 3 Pattern 2, with $r_{\textrm{error}}=0.344\,\hbox{m}$, and right is Run 5 Pattern 2, with $r_{\textrm{error}}=0.05\,\hbox{m}$. The swarm on the left has not converged on a single frame of reference, and at this point in pattern formation, the two groups of robots are too far apart to communicate

**Fig. 11:**
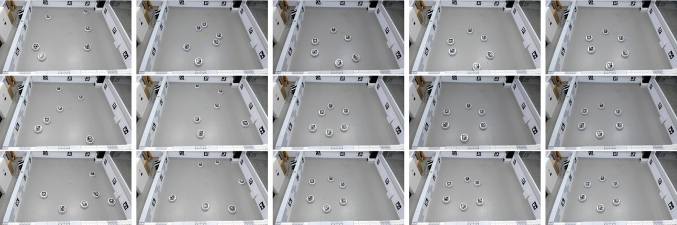
Formation of hexagon arrangement by DOTS robots repeated three times, frames from Run 1

We then look at the performance of pattern formation. Firstly, for the pattern to form correctly, the DSA shared reference frame needs to have converged. Table 5 shows the mean deviation $r_{\textrm{error}}$ in metres for each pattern in each run. In Sect. 3.2.1 we define convergence as $2\sigma _{\textrm{position}}$ which, in worse case with our distance dependent alteration to position sensing uncertainty, is 0.12 m. Only eight of the 15 pattern formation attempts meet this, although this criteria assumes unbiassed odometry measurement. It is clear that Run 3, Patterns 1 and 2 are significantly worse, and upon examination, in each case the swarm has two disconnected graphs; with two and four robots in each, and with the distance sufficiently far apart that no messages can pass between them to bring about convergence. The best and worst convergences are shown in Fig. 10, which are annotated frames from videos of the runs. Snapshots from Run 1 are shown in Fig. 11 to illustrate the pattern formation process. A video of all runs is available at https://youtu.be/6ZwQHJnqL7M, with annotation of the robot ground truth and the DSA frames.

Figure 9 right shows data from all pattern formation attempts except for Run 3, Patterns 1 and 2, and Table 6 summarises the vertex position error defined as:

13 $$\begin{aligned} v_{error} = \frac{1}{6\cdot n_{pats}} \sum _{p \in pats} \sum _{i=1}^6 {|r^i_{position}-r^i_{ideal}|} \end{aligned}$$

Overall $v_{\textrm{error}}= 0.084\,\hbox{m}$. Robots 2 and 6 have significantly worse $v_{\textrm{error}}$, and this would be expected given the characteristics of visual receptive field of the robots. Each cannot sense either of their neighbours. Despite this, because their neighbours *can* observe them, make outward-facing factors that connect to their local fragments of the factor graph, and thus initiate exchanges of GBP messages to them, they are still able to converge to within $0.12\,\hbox{m}$ of ideal position.

We have done very little tuning to the uncertainty assigned to the odometry and relative position measurements, and it is likely that the odometry uncertainty is too low, as well as failing to capture velocity differences in the robots; the effective wheel diameter varies from nominal resulting in different robots having different biasses, which are not currently captured by calibration. Despite the non-optimal uncertainty model, and the approximations of applying the 2D linear system, the system still works surprisingly well.

### Discussion

It is important to note that both DSA-KE (Knowledge Enhancement) and DSA-SF (Shape Formation) are simple algorithms that serve to illustrate the possibilities that distributed spatial awareness can offer for swarm algorithms. The behaviour of the GBP message passing algorithm underlying the DSA shared reference frame is robust across parameters and the computational cost per robot of maintaining its local factor graph is low, of the order of a few hundred floating point operations per second, and the communications likewise is low cost, being a few hundred bytes per second. This is within reach of even cheap microcontrollers.

There are some limitations to this work, particularly the use of linear factors in $\mathbb {R}^2$. As we mention below, we intend to move to $\textrm{SE}(2)$. Implementing the algorithm updates to support this is necessary to generalise to real-world applications. Likewise, the current assumption of time synchronicity is unnecessary for the general GBP algorithm, one simple approach we are considering is to tag outgoing messages with their local age; the receiving robot can allocate them to its closest equivalent age node. The convergence time proxy $t_{\textrm{convproxy}}$ requires knowledge of the size of the swarm, we are investigating unique encounter rates and possible swarm size consensus approaches to remove this limitation. Our shape-formation algorithm is extremely simple, and will fail in environments with obstacles, but this was to demonstrate the utility of the DSA shared reference frame. More robust algorithms can be constructed on this framework.

Successfully transferring DSA to real robots, without making alterations to the algorithm beyond slight changes to the uncertainty model further demonstrates the robustness of DSA to quite large deviations from the more idealised simulator. Even with gaps in the robot visual perception effectively blinding two of the six robots in the hexagon patten, the swarm collectively was still able to perform well. The resilience of the distributed factor graph and message passing underlying DSA is encouraging for future real-world deployment.

## Conclusion

We use exploratory movement, local observation, and distributed factor graph construction, combined with GBP evaluation, to give a global sensing ability to a swarm system without violating the core tenet of decentralisation, and with low per-robot computation and communication cost.

Using two simple algorithms that make use of this new distributed spatial awareness ability, we show the possibilities. Moving DSA to real robots demonstrates potential applicability and robustness beyond the lab. Applying some of the automatic design techniques commonly used for swarm controllers to systems using DSA is interesting; evolution often discovers non-obvious uses of new abilities. We consider GBP to be a powerful technique to infer global knowledge within a completely distributed paradigm, and wish to bring other items of state and knowledge within this overarching framework; it will be interesting to compare some more traditional swarm consensus algorithms with GBP, many best-of-n swarm algorithms rely on local message passing Valentini et al. (2017), perhaps suggesting deeper similarities.

We look forward to exploring new swarm algorithm possibilities that may combine DSA acquired knowledge with other swarm techniques. The current system relies on graph construction happening in synchronisation at regular timesteps, but we don’t see this as necessary and plan to remove this restriction, e.g. by estimating between states at time of observation. Finally, we are planning to move beyond the restrictions of pure 2D linear systems for further work on our DOTS robots moving towards a fully decentralised swarm logistics demonstration.

## Data Availability

No datasets were generated or analysed during the current study.

## Footnotes

1 The local reference frame of tag is located at centre of tag, with *x*-right, *y*-up, *z*-outward.

## References

[CR1] Agarwal, S., Mierle, K., & Team, T. C. S. (2023). Ceres solver. https://github.com/ceres-solver/ceres-solver

[CR2] Bickson, D., Shental, O., & Dolev, D. (2008). Distributed Kalman filter via Gaussian belief propagation. In: *2008 46th annual allerton conference on communication, control, and computing* (pp. 628–635).

[CR3] Birattari, M., Ligot, A., Bozhinoski, D., Brambilla, M., Francesca, G., Garattoni, L., & Stützle, T. (2019). Automatic off-line design of robot swarms: A manifesto. *Frontiers in Robotics and AI,**6*, 59. 10.3389/frobt.2019.00059 33501074 10.3389/frobt.2019.00059 PMC7806002

[CR4] Carrillo-Zapata, D., Sharpe, J., Winfield, A. F. T., Giuggioli, L., & Hauert, S. (2019). Towards controllable morphogenesis in large robot swarms. *IEEE Robotics and Automation Letters,**4*(4), 3386–3393. 10.1109/LRA.2019.2926961

[CR5] Catto, E. (2009). Box2D: A 2D physics engine for games. http://box2d.org/

[CR6] Crosscombe, M., Lawry, J., Hauert, S., Homer, M. (2017). Robust distributed decision-making in robot swarms: Exploiting a third truth state. In: T. Maciejewski (Ed.), *2017 IEEE/RSJ international conference on intelligent robots and systems (IROS)* (pp. 4326–4332). Vancouver, Canada.

[CR7] Davison, A.J., & Ortiz, J. (2019). FutureMapping 2: Gaussian belief propagation for spatial AI. arXiv:1910.14139.

[CR8] Dellaert, F., & Contributors, G. (2022). borglab/gtsam. Georgia Tech Borg Lab. https://github.com/borglab/gtsam

[CR9] Dellaert, F., Kaess, M., et al. (2017). Factor graphs for robot perception. *Foundations and Trends® in Robotics*, *6*(1–2), 1–139. 10.1561/2300000043

[CR10] Du, J., Ma, S., Wu, Y.-C., Kar, S., & Moura, J. M. (2018). Convergence analysis of distributed inference with vector-valued gaussian belief propagation. *Journal of Machine Learning Research,**18*(1), 6302–6339. http://jmlr.org/papers/v18/16-556.html.

[CR11] Erdős, P., Rényi, A., et al. (1960). On the evolution of random graphs. *Publications of the*

[CR12] Jadbabaie, A., Lin, J., & Morse, A. S. (2003). Coordination of groups of mobile autonomous agents using nearest neighbor rules. *IEEE Transactions on Automatic Control,**48*(6), 988–1001.

[CR13] Jones, S., Milner, E., Sooriyabandara, M., Hauert, S. (2022). DOTS: An open testbed for industrial swarm robotic solutions. 10.48550/arXiv.2203.13809

[CR14] Jones, S., Studley, M., Hauert, S., & Winfield, A.F. (2016). Evolving behaviour trees for swarm robotics. In: R. Groß et al. (Eds.), *13th International symposium on distributed autonomous robotic systems (DARS 2016)*. London: Springer.

[CR15] Jones, S., & Hauert, S. (2023). Frappe: Fast fiducial detection on low cost hardware. *Journal of Real-Time Image Processing,**20*(6), 119. 10.1007/s11554-023-01373-w

[CR16] Jones, S., Milner, E., Sooriyabandara, M., & Hauert, S. (2020). Distributed situational awareness in robot swarms. *Advanced Intelligent Systems,**2*(11), 2000110. 10.1002/aisy.202000110

[CR17] Jones, S., Winfield, A. F., Hauert, S., & Studley, M. (2019). Onboard evolution of understandable swarm behaviors. *Advanced Intelligent Systems,**1*(6), 1900031. 10.1002/aisy.201900031

[CR18] Li, G., St-Onge, D., Pinciroli, C., Gasparri, A., Garone, E., & Beltrame, G. (2019). Decentralized progressive shape formation with robot swarms. *Autonomous Robots,**43*, 1505–1521. 10.1007/s10514-018-9807-5

[CR19] Liu, W., & Winfield, A. F. (2010). Modeling and optimization of adaptive foraging in swarm robotic systems. *The International Journal of Robotics Research,**29*(14), 1743–1760. 10.1177/027836491037513

[CR20] Milner, E., Sooriyabandara, M., & Hauert, S. (2022b). Swarm diffusion-taxis: Transport of spatial information for cooperative gradient-based navigation. *AROB-ISBC-SWARM 2022*.

[CR21] Milner, E., Sooriyabandara, M., & Hauert, S. (2022). Stochastic behaviours for retrieval of storage items using simulated robot swarms. *Artificial Life and Robotics,**27*(2), 264–271. 10.1007/s10015-022-00749-8

[CR22] Montijano, E., Cristofalo, E., Zhou, D., Schwager, M., & Saguees, C. (2016). Vision-based distributed formation control without an external positioning system. *IEEE Transactions on Robotics,**32*(2), 339–351.

[CR23] Murai, R., Ortiz, J., Saeedi, S., Kelly, P. H., & Davison, A. J. (2024). A robot web for distributed many-device localization. *IEEE Transactions on Robotics,**40*, 121–138. 10.1109/TRO.2023.3324127

[CR24] Oh, H., Shirazi, A. R., Sun, C., & Jin, Y. (2017). Bio-inspired self-organising multi-robot pattern formation: A review. *Robotics and Autonomous Systems,**91*, 83–100. 10.1016/j.robot.2016.12.006

[CR25] Olfati-Saber, R., Fax, J. A., & Murray, R. M. (2007). Consensus and cooperation in networked multi-agent systems. *Proceedings of the IEEE,**95*(1), 215–233.

[CR26] Ortiz, J., Evans, T., & Davison, A.J. (2021). A visual introduction to Gaussian belief propagation. arXiv:2107.02308

[CR27] Ortiz, J., Pupilli, M., Leutenegger, S., & Davison, A.J. (2020). Bundle adjustment on a graph processor. In: *Proceedings of the IEEE/CVF conference on computer vision and pattern recognition* (pp. 2413–2422).

[CR28] Pan, Z., Wang, D., Deng, H., & Li, K. (2019). A virtual spring method for the multi-robot path planning and formation control. *International Journal of Control, Automation and Systems,**17*, 1272–1282. 10.1007/s12555-018-0690-9

[CR29] Patwardhan, A., Murai, R., & Davison, A. J. (2023). Distributing collaborative multi-robot planning with gaussian belief propagation. *IEEE Robotics and Automation Letters,**8*(2), 552–559. 10.1109/LRA.2022.3227858

[CR30] Pearl, J. (1982). Reverend bayes on inference engines: A distributed hierarchical approach. In: *AAAI’82: Proceedings of the second AAAI conference on artificial intelligence* (pp. 133–136). AAAI Press.

[CR31] Pitonakova, L., Winfield, A., & Crowder, R. (2018). Recruitment Near Worksites Facilitates Robustness of Foraging E-puck Swarms to Global Positioning Noise. *2018 IEEE/RSJ International Conference on Intelligent Robots and Systems (IROS)* (pp. 4276–4281).

[CR32] Reynolds, C.W. (1987). Flocks, herds and schools: A distributed behavioral model. *ACM SIGGRAPH Computer Graphics* (Vol. 21, pp. 25–34).

[CR33] Rubenstein, M., Ahler, C., & Nagpal, R. (2012). Kilobot: A low cost scalable robot system for collective behaviors. L. Parker (Ed.), *IEEE International Conference on Robotics and Automation (ICRA 2012)* (pp. 3293–3298). St. Paul, MN, USA.

[CR34] Rubenstein, M., Cornejo, A., & Nagpal, R. (2014). Programmable self-assembly in a thousand-robot swarm. *Science,**345*(6198), 795–799. 10.1126/science.1254295 25124435 10.1126/science.1254295

[CR35] Sabattini, L., Secchi, C., & Fantuzzi, C. (2011). Arbitrarily shaped formations of mobile robots: Artificial potential fields and coordinate transformation. *Autonomous Robots,**30*, 385–397. 10.1007/s10514-011-9225-4

[CR36] Schranz, M., Umlauft, M., Sende, M., & Elmenreich, W. (2020). Swarm Robotic Behaviors and Current Applications. *Frontiers in Robotics and AI,**7*, 36. 10.3389/frobt.2020.00036 33501204 10.3389/frobt.2020.00036 PMC7805972

[CR37] Slavkov, I., Carrillo-Zapata, D., Carranza, N., Diego, X., Jansson, F., Kaandorp, J., & Sharpe, J. (2018). Morphogenesis in robot swarms. *Science Robotics,**3*(25), eaau9178. 10.1126/scirobotics.aau9178 33141694 10.1126/scirobotics.aau9178

[CR38] Soares, J.M., Navarro, I., & Martinoli, A. (2016). The khepera iv mobile robot: performance evaluation, sensory data and software toolbox. *Robot 2015: second iberian robotics conference* (pp. 767–781).

[CR39] Stolfi, D. H., & Danoy, G. (2024). Evolutionary swarm formation: From simulations to real world robots. *Engineering Applications of Artificial Intelligence,**128*, Article 107501.

[CR40] Su, Q., & Wu, Y.-C. (2015). On Convergence Conditions of Gaussian Belief Propagation. *IEEE Transactions on Signal Processing,**63*(5), 1144–1155. 10.1109/TSP.2015.2389755

[CR41] Talamali, M. S., Bose, T., Haire, M., Xu, X., Marshall, J. A., & Reina, A. (2020). Sophisticated collective foraging with minimalist agents: A swarm robotics test. *Swarm Intelligence,**14*(1), 25–56. 10.1007/s11721-019-00176-9

[CR42] Tzoumas, G., Pitonakova, L., Salinas, L., Scales, C., Richardson, T., & Hauert, S. (2023). Wildfire detection in large-scale environments using force-based control for swarms of UAVs. *Swarm Intelligence,**17*(1–2), 89–115. 10.1007/s11721-022-00218-9

[CR43] Valentini, G., Ferrante, E., & Dorigo, M. (2017). The Best-of-n Problem in Robot Swarms: Formalization, State of the Art, and Novel Perspectives. *Frontiers in Robotics and AI,**4*, 9. 10.3389/frobt.2017.00009

[CR44] Weiss, Y., & Freeman, W. (1999). Correctness of Belief Propagation in Gaussian Graphical Models of Arbitrary Topology. *Advances in Neural Information Processing Systems 12 (NIPS 1999)* (Vol. 12). 10.1162/089976601750541769 11570995

[CR45] Winfield, A.F. (2009). Foraging robots. *Encyclopedia of Complexity and Systems Science* (pp. 3682–3700). Springer.

